# Are Movement Disorders and Sensorimotor Injuries Pathologic Synergies? When Normal Multi-Joint Movement Synergies Become Pathologic

**DOI:** 10.3389/fnhum.2014.01050

**Published:** 2015-01-06

**Authors:** Marco Santello, Catherine E. Lang

**Affiliations:** ^1^Neural Control of Movement Laboratory, School of Biological and Health Systems Engineering, Arizona State University, Tempe, AZ, USA; ^2^Program in Physical Therapy, Program in Occupational Therapy, Department of Neurology, Washington University School of Medicine in St. Louis, St. Louis, MO, USA

**Keywords:** coordination, degrees of freedom, stroke, dystonia, carpal tunnel syndrome

## Abstract

The intact nervous system has an exquisite ability to modulate the activity of multiple muscles acting at one or more joints to produce an enormous range of actions. Seemingly simple tasks, such as reaching for an object or walking, in fact rely on very complex spatial and temporal patterns of muscle activations. Neurological disorders such as stroke and focal dystonia affect the ability to coordinate multi-joint movements. This article reviews the state of the art of research of muscle synergies in the intact and damaged nervous system, their implications for recovery and rehabilitation, and proposes avenues for research aimed at restoring the nervous system’s ability to control movement.

## Introduction

The term “synergy” – from the Greek *synergia* – means “working together.” The concept of multiple elements working together toward a common goal has been extensively used to develop experimental approaches and analytical techniques to understand how the central nervous system (CNS) controls movement. A classic definition of synergy is “a collection of relatively independent degrees of freedom that behave as a single functional unit” [for review, see Turvey (2007)]. As noted in a recent review, this very definition of “synergy” can have different implications in terms of underlying mechanisms depending on the scale of the system at which it is applied, e.g., groups of motor units, muscles, and/or joints (Santello et al., 2013). However, one of the most widely studied and informative domains for identifying synergies has been muscle activity, quantified as patterns of interference electromyographic (EMG) activity recorded simultaneously across multiple muscles. This research has provided important insights into the modular nature of control of a variety of movements by identifying multi-muscle EMG patterns, i.e., *muscle synergies* [for review, see Ting and McKay (2007) and Bizzi et al. (2008)]; for discussion on the modular control of movement from an evolutionary perspective, the reader is referred to a recent review by Lacquaniti et al. (2013). Specifically, these muscle synergies have been defined as “building blocks” of complex movements that can be flexibly combined when performing different tasks, or the same task performed in different conditions, by modulating the timing and/or amplitude of EMG activity of individual muscles [for review, see d’Avella and Lacquaniti (2013)].

The quest for synergies at different levels of the system has led to the development of several approaches to quantify the structure and number of synergies in a variety of tasks, including the modulation of hand posture to object geometry [e.g., principal components analysis (Santello et al., 1998, 2002) and singular value decomposition (Mason et al., 2001)], digit force coordination during force production tasks [uncontrolled manifold analysis, UCM: for review, see Latash et al. (2002); see also Latash and Anson (2006)] for application of UCM analysis to study the effects of neurological disorders and aging on motor coordination), multi-digit force coordination during prehension [hierarchical organization of grasp variables and virtual finger; for review, see Zatsiorsky and Latash (2004)], and coordination of activation across multiple muscles [non-negative matrix factorization: for review, see Tresch et al. (2006)]. For more details on how these techniques have been applied to a large variety of motor tasks and their interpretation, the reader is referred to a recent review on the neural bases of hand synergies (Santello et al., 2013). Importantly, how synergies are defined or conceptualized may or may not account for how synergies are built and retrieved, or how flexible they might be [for a detailed overview of theoretical frameworks underlying synergies, the reader is referred to Latash (2008) and Santello et al. (2013)]. However, it has been proposed that the role of synergies may be to map high-level goals to multi-muscle activation patterns, as suggested by their sensitivity to specific task performance goals (Ting and McKay, 2007; Tresch and Jarc, 2009). Furthermore, even though several mechanisms could potentially be involved in coupling the activity of multiple muscles (e.g., reflexes, supraspinal networks, and/or spinal modules driven by supraspinal drive), these mechanisms need not be mutually exclusive [for review, see Bizzi and Cheung (2013) and Giszter and Hart (2013)]. Importantly, a common denominator across many definitions and interpretations is that biomechanical and neural factors constrain the spatial and temporal coordination of groups of muscles and joints. The net effect of the interaction of these constraints is a reduction in the number of degrees of freedom that are, or can be, controlled during movement execution.

In the clinical domain, however, the term synergy is not applied uniformly. Over the years, “synergy” has been used in a variety of different ways, such that sometimes the same term represents different constructs or phenomena. Other times, different terms have been used to express the same construct or phenomenon. One of the earliest uses of the term “synergy” occurred at the beginning of the 20th century when Babinski intended to describe the pathological flexor reflex response caused by stimulation on the plantar surface of the foot in the presence of corticospinal tract damage, i.e., “the Babinski sign” (Van Gijn, 1978). Since then, “synergy” has been broadly used to describe abnormal, stereotypic movements, such as in Brunnstrom’s depiction of abnormal synergies after corticospinal system damage (Brunnstrom, 1966, 1970). In more recent times, the term “synergy” is still used (Cheung et al., 2012), along with other terms such as “abnormal coupling,” “abnormal coactivation” (Dewald et al., 1995), and “motor overflow” (Tung et al., 2011). Within the above-described framework of “muscle synergies,” the study of pathological synergies following stroke has identified these synergies as emerging from one or more phenomena affecting the system’s ability to flexibly combine a given set of muscles by modulating their timing and/or activity, thus resulting in the use of a smaller number of muscle synergies, or fractions of physiological synergies (Clark et al., 2010; Safavynia et al., 2011; Cheung et al., 2012; Bizzi and Cheung, 2013).

The present paper focuses on the effects of neurological disorders on the ability of the CNS to coordinate movement through synergies. Although many definitions of synergies have been proposed [for review, see Tresch et al. (2006) and Santello et al. (2013), for review], it is generally agreed that a common feature spanning across these multiple definitions is that the coordination of multiple variables (muscles or joints) occur within a lower dimensional space than the available number of dimensions involved in the task. We will use the term “synergy” to denote patterns of voluntary muscle activity and multi-joint coordination that – while emerging in a repeatable fashion across subjects and movement repetitions – reduce the dimensionality of the control space while maintaining a certain degree of flexibility in terms of spatial and/or temporal coupling to task requirements. The definition we use here is motivated by the need to use a “baseline” of physiological coordinated movements, which involve multi-degrees of freedom to which pathological synergies can be compared to. Pathological synergies can arise from both volitional and reflexive control of movement. By this definition, a pathological synergy could be a loss of coordinated muscle patterns (and therefore movement), a more fixed or constrained set of coordinated muscle patterns, or a smaller number of synergies identified in the physiological system, e.g., the contralateral limb to the side affected by stroke (Cheung et al., 2012). The neurological disorders discussed in subsequent sections can affect the repeatability with which a given movement can be executed, and/or the flexibility with which it can be adapted to different task demands or circumstances.

## Synergies in the Intact System

The interaction between biomechanical and neural constraints dictates how and whether multi-joint motion may occur. Although the anatomy of the nervous and muscular systems appear to favor distributed neural inputs to multiple muscles and motion at several joints (Schieber and Santello, 2004; Santello et al., 2013), the “holy grail” of motor neuroscience research on synergies remains determining the extent to which biomechanical constraints shape motor commands that underlie the coordination of multi-joint movements. For example, when considering the neural control of the hand, biomechanical constraints such as tendons spanning three joints of the index finger would predict that activation of flexor muscles would produce torques and motion at all of the digital joints. However, this particular movement pattern is not obligatory. We can flex the index finger about one joint only, e.g., metacarpal–phalangeal joint, while blocking flexion of the proximal and distal inter-phalangeal joints. The former scenario occurs when we intend to firmly grasp an object, whereas the latter scenario occurs when we use the finger in slight flexion of all joints to type or press on the display of a smart phone. These examples underscore a critical issue: the CNS can take advantage of biomechanical constraints to generate multi-joint movements, but maintains *some ability* to override them depending on the demands of the task. Hence, in the intact system, such flexibility is crucially important not only to enable a wide variety of motor behaviors but also to allow movement patterns to flexibly adapt to different task conditions, e.g., walking on hard floor versus sand, or grasping a cylinder by flexing all fingers versus using only the thumb and index fingertip for dexterous manipulation.

The extent to which patterns of coordination emerge or exist in the activation of multiple muscles and multi-joint movements or forces has been extensively studied across many tasks, including single digit force production (Valero-Cuevas, 2000), multi-digit force production tasks (Zatsiorsky et al., 2000), hand shaping (Santello et al., 1998; Mason et al., 2004; Vinjamuri et al., 2007), multi-digit grasping (Santello and Soechting, 2000; Rearick et al., 2003; Zatsiorsky et al., 2003), typing (Soechting and Flanders, 1997), postural control (Lockhart and Ting, 2007), and gait (Ivanenko et al., 2004). First, this requires a balance of activation of the intrinsic muscles inside the hand and extrinsic muscles in the forearm. In addition, there is a coexistence of flexibility and repeatability of coordination patterns found across these tasks. For example, single digit force production is characterized by consistent correlations in EMG amplitude from all index finger muscles. At the same time, however, a certain degree of independent control of individual finger muscles has also been observed when examining the structure of task-relevant and -irrelevant variability (Valero-Cuevas et al., 2009). Similarly, consistent patterns of multi-digit force coordination are found when holding an object statically against gravity (Santello and Soechting, 2000; Rearick and Santello, 2002). Such patterns of synchronous force relations, however, are not found when the same forces are exerted on the same object when subjects are not required to prevent object slip (Rearick et al., 2003). A synergy-based framework has also been used to describe the coordination of multiple trunk and leg muscles during gait. This has been deduced because only five factors have been extracted from principal component analysis to account for the spatiotemporal variability in EMG activity elicited by walking at different speeds and loads (Ivanenko et al., 2004).

## A Theoretical Framework for Synergistic Control of Movement

A recent review of the literature on synergies underlying the control of the hand led to a theoretical framework, which emphasizes the role of spinal circuitry and the interplay of excitatory and inhibitory inputs from spinal premotor neurons in implementing synergistic control of hand muscles (Santello et al., 2013). Briefly, pools of premotor neurons are defined as a dynamical system capable of changing “state,” where each state corresponds to specific patterns of muscle activation or synergies. The shape of the potential function of the dynamics of the system is controlled by descending motor commands. A given pattern of muscle activation would result from the convergence of inputs from pools of inhibitory and excitatory premotor neurons to alpha-motor neurons. It should be noted that this framework, at this stage, is purely theoretical. Therefore, its validation and the extent to which it can be integrated with existing theoretical frameworks [see Santello et al. (2013) for review] remain to be established experimentally. Nevertheless, this theoretical framework does highlight important features that are found across many tasks involving upper or lower limb muscle synergies, such as the ability to learn new synergies and the extent to which a given synergy might appear to be “fixed” or “flexible.” For example, extensive motor practice would lead to adaptation of the premotor circuitry that would generate the same synergy in a consistent fashion despite variability or noise in the spatiotemporal patterns of motor commands, hence appearing as a “fixed” synergy. Conversely, a “flexible” synergy would result from several clusters of premotor circuitry that can be recruited with similar likelihood for similar sets of descending and ascending inputs [flat potential field in Figure 4 in Santello et al. (2013)]. Common to this definition of synergies and earlier definition of muscle synergies is the notion that the organization of spinal circuitry has evolved to combine spinal modules for controlling multiple muscles in a way that reduces the large dimensionality afforded by the number of available muscles.

Importantly, this framework emphasizes the role of sensory feedback in affecting the net input of premotor neuron pools to alpha-motor neurons as well as in signaling unexpected events to higher levels of the motor system. In the present review, we will use this framework as a model for describing the effects of central and peripheral pathologies on the CNS’ ability to implement synergy-based control. Note that this theoretical framework is compatible with the muscle synergy model proposed by Bizzi and Cheung (2013). However, the former framework does not provide a formal definition of “modules” and focuses on premotor neuron networks as the main “node” of the system responsible for creating and selecting muscle synergies. Despite these differences, selective disruption of premotor circuitry involved in the selection of specific muscle synergies would predict the same behavioral outcomes that have been described in stroke patients, i.e., merging and/or fractionation of physiological muscle synergies.

## Effects of Various Pathologies on the Presence and Control of Synergies

**Figure 1:**
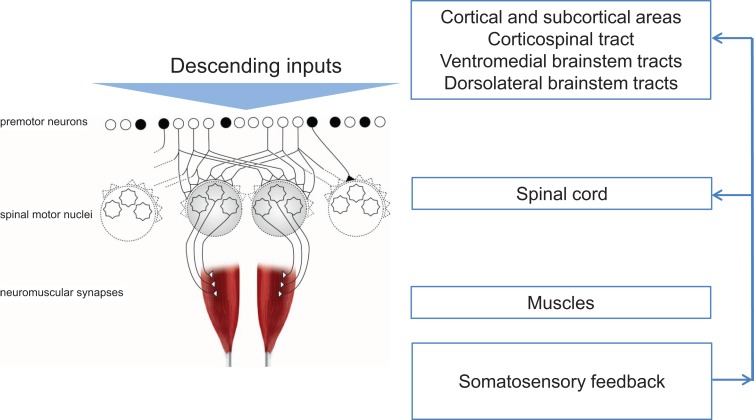
**Conceptual representation of physiological synergies (left) with anatomical labels (right)**. Black circles denote inhibitory premotor neurons. Adapted from Santello et al. (2013).

Injury or disease to the nervous system can affect the physiological synergies discussed above, resulting in pathological synergies for movement control. In the following sections, this review discusses how damage to various parts of the nervous system influences synergistic control. Figure 1 shows a conceptual representation of physiological synergies on the left, with anatomical labels on the right. Two conditions presented here alter the descending inputs from cortical and subcortical structures; these are stroke and focal hand dystonia. Two other conditions, spinal cord injury and carpal tunnel syndrome (CTS), disrupt the physiological activation of premotor and spinal motor nuclei and somatosensory feedback, respectively. These four conditions are not intended to be an all-inclusive list of conditions that result in pathological synergies, but rather they were selected based on sufficient available literature. Within each condition, we discuss implications for recovery and motor rehabilitation.

### Stroke

The majority of published work on pathological synergies has been done in people with stroke. A stroke occurs when a blood vessel in the brain is blocked (ischemic stroke), or ruptured (hemorrhagic stroke), resulting in lack of blood flow and oxygen supply to a part of the brain, causing neurons to die. The types and degree of impairment following a stroke depend upon which areas of the brain are damaged and the extent of that damage (Ninds, 2013). Stroke survivors can experience difficulty with movement, language, cognition, somatosensation, vision, and other functions (Gresham et al., 1979; Go et al., 2013). Whereas motor deficits, particularly paresis, are the most common, people often experience deficits in multiple domains, because the disruption of blood flow does not respect functional anatomic boundaries. The upper limb is often more impaired than the lower limb when the lesion occurs within the middle cerebral artery distribution (the most common arterial distribution for stroke). In contrast, the lower limb is more impaired than the upper limb when the stroke occurs within the anterior cerebral artery distribution. Early clinical observations of pathological synergistic control noted that, as volitional movement emerged over the course of stroke recovery, single joint actions did not occur in isolation, but instead occurred with actions of the other joints of the limb (Twitchell, 1951; Brunnstrom, 1970). In addition, volitional movements observed in supine and sitting were often unavailable when standing up against gravity, as it requires a greater generation of forces to initiate limb movement when having to overcome gravitational forces.

**Figure 2:**
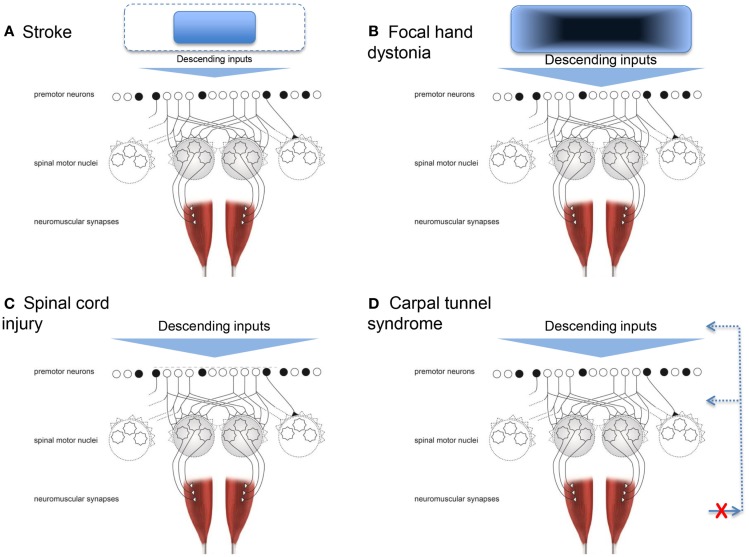
**Conceptual representations of how four conditions can result in pathological synergies**. **(A)** Stroke, where the neural substrate from which the descending inputs arise is reduced. **(B)** Focal hand dystonia, where the neural substrate the descending inputs arise from has abnormal inhibitory circuitry. **(C)** Spinal cord injury, where the premotor and spinal motor neurons are damaged. **(D)** Carpal tunnel syndrome, where the somatosensory feedback for synergistic control is disrupted. Adapted from Santello et al. (2013).

Neuronal death caused by stroke affecting the motor cortex and its output, the corticospinal tract, essentially limits the cortical neural substrate available for physiological synergistic control. In other words, the range and flexibility of the descending command is limited. This is conceptually depicted in Figure 2A. With a smaller number of cortical cells available (or connected), the control options are reduced. Specifically, the potential function we described above would lose its ability to be shaped by descending input as well as the integration of descending and ascending inputs. As a result, stable but fewer patterns of muscle activity would arise, thus resulting in a reduced ability to adapt to task demands. The first quantifications of these pathological synergies reported muscle activation patterns at the shoulder and elbow that were no longer direction specific, and an inability to isolate activation to a particular muscle (Bourbonnais et al., 1989; Dewald et al., 1995). For example, when attempting to activate shoulder abductor muscles, subjects also activated elbow flexor muscles. The opposite also occurred, such that when attempting to activate elbow flexor muscles, subjects also activated shoulder abductor muscles. Kinetic output in the presence of less muscle isolation and more coactivation resulted in force production that was less focal with abnormal coupling between shoulder and elbow actions (Dewald and Beer, 2001; Tung et al., 2011). Similarly, the kinematic output presented as a reduced ability to move in all directions and smaller available workspace for the shoulder and elbow (Beer et al., 2007).

The pathological synergies poststroke extend beyond the shoulder and elbow, spreading across the entire upper limb (Kamper and Rymer, 2001; Lang and Beebe, 2007; Ohn et al., 2013). Small, isolated strokes to the primary motor cortex or to the corticospinal tract can be sufficient enough to cause a loss of selective finger muscle activation, such that the fingers tend to move together instead of as individuated movements (Lang and Schieber, 2003, 2004). As the lesions become larger and cause neural damage beyond these areas, the overall picture of loss of fractionated finger movements is similar (Raghavan et al., 2006). Even in people who are severely affected poststroke, one can see abnormal synergistic control, as indicated by coactivation and more stereotypic movement patterns across the entire arm (Lan et al., 2011; Miller and Dewald, 2012). The degree of pathological synergy (i.e., loss of fractionated movement) is consistently correlated with the loss of functional upper limb ability, regardless of the specific subpopulation studied and/or the methods for quantifying synergistic movements and function (Bourbonnais et al., 1989; Lang and Schieber, 2003, 2004; Raghavan et al., 2006; Lang and Beebe, 2007; Beebe and Lang, 2008; Miller and Dewald, 2012; Ohn et al., 2013).

The number of non-obligatory upper limb synergies available for movement function after stroke is reduced, with an emerging appreciation that the numerous, physiological pre-stroke synergies may be merged to only a few available pathological synergies poststroke (Cheung et al., 2012; Roh et al., 2013). In these studies, EMG is recorded from multiple limb muscles. The EMG signal is decomposed and then re-assembled into factors (i.e., synergies) using non-negative matrix factorization. The premise here is that the quantifiable synergies extracted represent the underlying building blocks of the motor system (Tresch et al., 2002; Flash and Hochner, 2005; Bizzi et al., 2008). The decision about the final number of synergies to select is determined by the amount of variance explained from the EMG signal; common thresholds are 80–90% of variance explained. Interestingly, pathological synergies are apparent relatively early after stroke (Beebe and Lang, 2008; Ellis et al., 2011). These synergies are most predominant when the patient is required to move against gravity. In more mildly affected individuals, the synergies can improve quickly over time (Ellis et al., 2011). With people who are more moderately affected, the presence of some merged and some partial pre-stroke synergies later poststroke may reflect a compensatory control scheme to enable functional movement capabilities (Cheung et al., 2012). Increasing effort required for movement, often tested with increasing gravitational load on the upper limb, results in more pronounced (worsened) abnormal control, as indicated by more coactivation and a reduced ability to produce fractionated movement out of the pathological synergy pattern (Beer et al., 2007; Ellis et al., 2007).

The lower limb and its movements are similarly affected by the reduced neural substrate poststroke, leading to reduced control options and fewer, more stereotypic movement patterns. Neurologically intact individuals use variable activation of four motor synergies (or modules) to produce gait (Clark et al., 2010). The four synergies roughly correspond to (1) extensor activity at the hip and knee during early stance to support body weight; (2) flexion activity at the knee and ankle (plantar flexion) during late stance for forward propulsion of the body and swing initiation; (3) flexor activity at the ankle (dorsiflexion) knee, and hip during early stance to eccentrically decelerate the limb segments, and through to early swing to allow the foot to clear the ground; and (4) flexion activity at the knee and extensor activity at the hip during late swing into early stance to decelerate the limb segments and propel the body forward during stance (Neptune et al., 2009). Poststroke individuals tend to have access to fewer motor synergies for gait control than neurologically intact individuals (Clark et al., 2010; Allen et al., 2013). Abnormal coupling between leg muscles is not only seen during gait but also during instructed volitional activation, such as during instructed isometric hip adduction (Krishnan and Dhaher, 2012). Interestingly, activation of limited synergies may be the primary means to activate some lower limb muscles, as indicated by lower limb muscle activation (tibialis anterior) that occurs during gait but not during instructed volitional activation (Bowden et al., 2010). Thus, literature from both the upper limb and the lower limb contributes to the current understanding of pathological synergies poststroke, despite the perceptions of relative differences in the importance of various neural control structures, i.e., motor cortex for upper limb control versus spinal cord for lower limb control.

The implications of the pathological synergies poststroke on recovery and motor rehabilitation are complex. As implied above, the presence of mild pathological synergy (or even the presence of intact physiological synergies) is an indicator of good recovery after stroke. The presence of strong pathological synergies is most often accompanied by other, key motor deficits, such as paresis, and collectively, these are indicators of poor recovery and loss of functional ability (Lang and Beebe, 2007; Lang et al., 2013).

Some of the earliest rehabilitation approaches were designed to avoid pathological synergistic movements and to retrain only normal movement patterns (Brunnstrom, 1970). Unfortunately, after many years of clinical trials, these approaches have not been shown to improve motor impairments and function for people with stroke (Veerbeek et al., 2014). This may be because pathological synergies, once present beyond the mild stage, are not amenable to change, or because the overall approach tested was a more conceptual, and not an explicit, rigorous attempt to “untrain” the pathological synergies.

Two modern reports have specifically addressed synergistic control after stroke. In the first, motor training was explicitly targeted toward reducing coupling of affected shoulder and elbow muscles (Ellis et al., 2005). After 8 weeks of training, subjects with moderate-to-severe stroke could produce shoulder and elbow torques that were less coupled and more varied than the pre-training stereotypic patterns, i.e., an improvement in the pathological synergy. However, improvements were small, and their clinical relevance was not clear. In the second study, the motor training was not explicitly targeted toward modifying synergistic control, but instead targeted at regaining walking function (Routson et al., 2013). After 12 weeks of training, subjects with mild-to-moderate stroke had leg motor synergies for walking that more closely resembled the synergies of neurologically intact individuals and had an increased number of available synergies to access during walking. Improvements in walking function were clinically meaningful as well.

Further study into the modifiability of pathological synergistic control poststroke is needed. Specifically, factors such as stroke severity and body parts trained make it difficult to determine if intervention approaches specifically targeting abnormal synergistic control will eventually produce better outcomes. Caution is warranted, however, on the issue of specificity of training in relation to functional outcomes because neurorehabilitation has a long history of targeting specific impairments, where the impairment-based approaches have led to improvements in those impairments, but not to improvements in daily function (Duncan et al., 2005; Teasell et al., 2008).

### Focal hand dystonia

Focal dystonia alters synergistic control in a manner different to that of stroke. Dystonia refers to a group of movement disorders characterized by coactivation of agonist and antagonist muscles, leading to abnormal, twisting, end range repetitive postures (Fahn, 1988). This review focuses on studies of focal hand dystonia because this is the specific dystonic population in which investigation of upper limb movement control is most common. The causes of focal hand dystonia are multifactorial and include genetics (Altenmuller and Jabusch, 2009; Neychev et al., 2011; Jinnah et al., 2013). Even for people with a genetic risk for dystonia, all individuals will not go on to develop dystonia. The genotype can lead to the phenotype of dystonia in the presence of other factors, such as stress, anxiety, heavy repetitive hand use, and trauma (Altenmuller and Jabusch, 2009). Most focal hand dystonias are task specific and arise from many years with long hours of repetitive activity (Torres-Russotto and Perlmutter, 2008b). For a review of similarities and differences across the adult-onset focal dystonias, see Jinnah et al. (2013). Two common forms arise from extended periods of writing (writer’s cramp) or playing a musical instrument (Torres-Russotto and Perlmutter, 2008a). Prevalent movement behaviors include tremor and abnormal muscle activations during the dystonia-inducing task, i.e., loss of ability to flexibly adapt the coordination pattern (Jinnah et al., 2013). For example, a musician may be unable to isolate ring and little finger movements while playing the violin, and instead activate multiple muscles of the wrist, hand, and forearm simultaneously. The pathological synergy is usually apparent only in one specific task early in the course of the disease, but spreads to other movements, and even up the entire limb, as the condition progresses without intervention. The specific pathological synergies, or patterns of abnormal movement, are not similar across individuals with hand dystonia (e.g., writer’s cramp, musician’s cramp, keyboarder’s cramp). For example, while patients with writer’s cramp may excessively squeeze the pen and increase the pressure down with writing, these patients do not necessarily exert excessive force with grasping and manipulating other objects (Hermsdorfer et al., 2011; Schneider et al., 2014). Across the variations of focal hand dystonia, deficits are not seen during basic daily movements, such as reach-grasp-lift (Odergren et al., 1996; Nowak et al., 2013), likely because the planned movement does not require the specific, overused pattern of premotor activation. The fact that the pathological synergies are observed only (at least at first) during the dystonia-inducing task is distinctly different from the pathological synergies seen poststroke and spinal cord injury, where they are present for nearly all movements. In the above example of stroke, pathological synergistic control was due to loss of corticospinal system neurons, i.e., observed movements are a result of what is left intact. In contrast, in focal hand dystonia, the motor cortex and pathways are intact, but the organization within and among structures is abnormal (Hallett, 2011; Altenmuller and Muller, 2013). Thus, observed movements emerge from the disrupted cortical and subcortical organization; this is depicted conceptually in Figure 2B. While this conceptualization is rather generic, it is a theoretical model, not a computational one. Future studies could test this theoretical model by quantifying the number and nature of synergies using methods applied poststroke (Cheung et al., 2012; Roh et al., 2013) to people with focal hand dystonia and other conditions resulting in disordered cortical and subcortical organization (e.g., Parkinson disease). Specifically for focal hand dystonia, the extensive repetitive practice leading up to dystonia may result in highly sensitive and finely tuned premotor neurons, and over time, a degradation in somatosensory discrimination and cortical processing. Eventually, the descending command has changed sufficiently that the highly sensitive premotor neuronal shaping may be inappropriate. The framework would predict a larger number of abnormal synergies observed and that they may not be stereotypic in form.

A consensus in the field is that pathological synergistic control seen with focal hand dystonias arises from a loss of inhibition (Hallett, 2011; Quartarone and Hallett, 2013). The problem is essentially one of excessive movement, where the normal mechanisms that inhibit unwanted movements are disrupted. Inhibitory circuitry of multiple types (e.g., surround inhibition, short-interval cortical inhibition) are dysfunctional, resulting in coactivation, loss of selective activation, and thus, excessive movement that is unintended (Beck et al., 2008; Beck and Hallett, 2011; Quartarone and Hallett, 2013). Loss of inhibition occurs at the level of local circuits related to the control of movement (Moore et al., 2012) as well as across larger scale brain networks (Jin et al., 2011a,b). In addition to the loss of inhibition, people with focal hand dystonia also have a mild loss of somatosensation and altered sensorimotor integration (Bara-Jimenez et al., 2000a,b; Quartarone and Hallett, 2013), such that the afferent information is slightly inaccurate and not easily utilized to plan future movements. The loss of inhibition and mild somatosensory deficits are further compounded by evidence of maladaptive plasticity, where there is an exaggerated responsiveness to repetitive inputs (Hallett, 2011; Quartarone and Hallett, 2013).

It is not clear if the maladaptive plasticity is a unique feature versus one that arises from altered inhibitory circuits, diminished somatosensory information, or extended periods of repetitive task-practice. The answer likely lies somewhere in the middle, given that the degree of large-scale cortical network deficiencies is moderately related to duration of the disease (Jin et al., 2011b). Particularly relevant for distal upper limb control is the loss of surround inhibition at low force levels (Beck et al., 2009). Because the majority of precise task-specific movements involve low force levels, the greatest problems with synergistic movement control, i.e., a loss of selective muscle activation, occurs exactly at the time when the person desires precise control the most. This is opposite to the increasing pathological synergistic control seen with increasing forces and effort levels in people with stroke (see previous section).

Literature on recovery and motor rehabilitation of focal hand dystonia is relatively sparse. Focal hand dystonia does not resolve spontaneously, and people are often reluctant to give up the intensive dystonia-inducing actions (e.g., professional musicians do not want to stop playing their musical instrument). Numerous interventions have been tried, with each intervention aimed only at a piece of the pathology and resulting in inconsistent or limited success (Jinnah et al., 2013; van Vugt et al., 2014). A common treatment is injection of small amounts of botulinum toxin, in order to quiet the muscles. For addressing pathological synergies of the hand, the injection of botulinum toxin can be quite challenging as there are a large number of muscles involved and precise motor control is highly valued (Hallett et al., 2009). Even with EMG-guided injections toward the most affected muscles, results can be variable across patients and studies (Hallett et al., 2009; Jinnah et al., 2013), perhaps because the intervention is addressing the symptoms rather than the cause of the pathological synergy.

Other types of intervention for focal hand dystonia address the mechanisms of the pathological synergy. These interventions range from immobilization to various sensorimotor retraining strategies for the hand and individual digits (Candia et al., 1999, 2003; Zeuner and Hallett, 2003; Zeuner et al., 2005; Baur et al., 2009; Byl et al., 2009; Cogiamanian et al., 2009). These interventions target the maladaptive plasticity with the goal of facilitating flexibility of the pathological synergies. A more novel intervention is transcranial magnetic stimulation with the goal of increasing cortical inhibition (Cogiamanian et al., 2009; Quartarone, 2013). Combinations of these interventions are currently being explored. Given the complex mechanisms of this relatively uncommon condition, any conclusions regarding rehabilitation effectiveness for focal hand dystonia are premature based on the small sample sizes, limited patient compliance, and limited follow-up data.

### Spinal cord injury

Another population in which motor synergies are commonly studied is spinal cord injury (Giszter and Hart, 2013). Disruption to the motor system happens above and at the level of the premotor and spinal motor neurons (Figure 2C). Signs and symptoms after spinal cord injury occur because of damage to the ascending and descending pathways within the cord (traditionally referred to as upper motor neuron signs), and because of damage to the spinal inter- and motor neurons (lower motor neuron signs). Thus, damage may have a direct result on how the physiologic synergies are accessed and implemented *and* on whether or not they can be accessed at all (Shapkova and Schomburg, 2001; Zariffa et al., 2012), as well as the extent to which they can be modulated by afferent inputs (Figure 1).

Observations of pathologic synergies after spinal cord injury have been documented most commonly at the kinematic and EMG levels. The general consensus that arises from these observations is that physiological synergies are present and accessible at their most basic level (de los Reyes-Guzman et al., 2010; Kloosterman et al., 2010; Wu et al., 2011; Field-Fote et al., 2012), as long as the spinal motoneurons can still activate their muscles (Gronley et al., 2000; Koshland et al., 2005). Some synergistic patterns are changed to compensate for muscles that can no longer be activated (Lin et al., 2008; Jacquier-Bret et al., 2009). There is early evidence opening a debate as to whether the number of synergistic patterns (i.e., motor modules) available is reduced after spinal cord injury (Zariffa et al., 2012; Fox et al., 2013) versus whether the available synergistic patterns are just arranged differently (Ivanenko et al., 2003, 2009).

Recovery after spinal cord injury can be reasonably predicted based on the segmental level of injury to the cord and on whether or not the injury is clinically complete versus incomplete. For those with incomplete injury, substantial efforts have been made to help patients increase access to available synergistic patterns to promote recovery of walking and other functions (Harkema et al., 2012; Hubli and Dietz, 2013; Morawietz and Moffat, 2013). It is well documented that available synergistic patterns can still be modified in response to a variety of inputs (Harkema et al., 1997; Gordon et al., 2009; Dy et al., 2010). Indeed, a major focus of rehabilitation research for people with incomplete spinal cord injury is to find and optimize innovative methods to access and modify preserved synergies for functional gain (Harkema et al., 2012; Hubli and Dietz, 2013).

### Carpal tunnel syndrome

Dexterous hand control requires integration of sensory feedback with motor commands responsible for digit placement and force sharing among the digits. Another neuromuscular disorder that can affect synergistic control of digit forces is CTS. In this disorder, somatosensory feedback about the hand and hand movements is not readily available to optimize control of finger muscles (Figure 2D). CTS is a compression neuropathy of the median nerve. Mild CTS causes somatosensory deficits in the thumb, index, middle, and lateral half of the ring finger, but in severe cases median nerve compression can cause motor deficits primarily in the thumb. Mechanical compression of the median nerve over long periods of time can cause ischemic damage and/or changes in the nerve myelination, and consequently slowing of axonal conduction velocity, nerve block, and in severe cases axonal loss (Welford, 1972; Nora et al., 2004). CTS signs and symptoms include numbness, aching, and or burning on the volar aspect of the affected hand, somatosensory loss, weakness, hyporeflexia, and clumsiness. People with CTS often complain about difficulties in performing activities of daily living, such as dropping objects, or dexterous tool use.

Investigations of the effects of CTS on the coordination of finger movements indicate that people with CTS exhibit large across-trial variability in precision pinch movements (Gehrmann et al., 2008) as well as reach-to-pinch kinematics (Nataraj et al., 2014). CTS studies on the coordination of multi-digit forces have revealed consistent disruption of synergistic coordination of grip force and load force (normal and tangential to the grasp surface, respectively) as a function of object mass, mass distribution, and texture. Specifically, whereas healthy individuals are known to exert grip forces that are only slightly larger than the minimum required to prevent object slip (“safety margin”; Westling and Johansson, 1984), people with CTS tend to exert significantly and systematically larger grip force than controls when using a whole-hand grasp (Zhang et al., 2011, 2012; Afifi et al., 2012). The systematic force overshoot might represent a decrease of sensory feedback and the development of a compensatory strategy to minimize risks of dropping the object. This strategy would compensate for deficits in tactile sensation and, possibly, sensorimotor integration. Importantly, however, the above CTS-induced impairment in the synergistic control of grip and load forces affects not only static grasp force production (e.g., holding an object against gravity) but also the *anticipatory* modulation of digit forces that occurs prior to the onset of object manipulation, e.g., object lift. Anticipatory grasp control relies on integrating sensorimotor memory of previous manipulation with online monitoring of sensory feedback [for review, see Johansson and Flanagan (2009)]. The “memory” component associated with anticipatory control is likely to be related to the reorganization of cortical sensory and motor areas associated with inflammation of peripheral tissues due to repetitive motor behaviors (Coq et al., 2009). As CTS impairs sensory function of a subset of the digits, one would expect that both the formation of sensorimotor memory *and* the integration of online sensory feedback would be affected. Specifically, people with CTS are less able than controls to use prior experience to scale digit forces in an anticipatory fashion to object weight (Zhang et al., 2011) and are less successful when distributing digit forces to prevent rotation of the object (Zhang et al., 2012). Another deficit in anticipatory control is the reduced ability to balance digit forces, resulting in unnecessary net moments at object lift onset when about to lift objects with a symmetrical center of mass (Zhang et al., 2013). When people with CTS are asked to lift an object with an asymmetrical mass distribution, they can learn to generate a compensatory moment and minimize object roll to the same extent as controls. However, multi-digit force coordination in controls fully exploited the available degrees of freedom to generate a compensatory moment, i.e., digit normal forces, tangential forces, and the net center of pressure on the finger side of the device at object lift onset and during object hold. In contrast, people with CTS modulated only the finger net center of pressure at object lift onset by modulating normal force sharing patterns while using the same normal and tangential forces across all object CMs. During object hold, however, people with CTS were able to modulate digit tangential force distribution to object CM. Therefore, although CTS did not affect a person’s ability to perform dexterous manipulation, it interfered with the modulation of specific grasp control variables. This phenomenon might be indicative of a lower degree of flexibility of the sensorimotor system in CTS to adapt to grasp task conditions, specific to the loss of sensation and strength following peripheral neuropathy.

As CTS is a compression neuropathy of the median nerve, rehabilitation interventions have focused on relieving the compression and its associated symptoms. Current best evidence suggests a multi-modal approach with steroid injections or other modalities to relieve inflammation, and splinting and exercise to improve wrist position and reduce compression (Piazzini et al., 2007; Page et al., 2012). Outcomes in rehabilitation clinical trials for CTS typically measure impairments such as pain symptoms or grip strength (Page et al., 2012), but do not examine the ability to flexibly adapt synergistic upper extremity movement patterns as a function of task demands and sensory inputs. To date, there are no longitudinal studies examining the effects of rehabilitation interventions aimed at modifying CTS-induced pathological multi-digit force synergies, e.g., non-zero net torque on the object at lift-off. Ongoing work is now quantifying how carpal tunnel release surgery, which allows for partial median nerve regeneration, might lead to restoration of physiological digit force coordination patterns (Santello et al., 2014).

## Some Additional Thoughts Across and between Conditions

From a historical perspective, pathological synergies have been readily identified after stroke and spinal cord injury. Focal hand dystonia and CTS have rarely been included in the list of conditions causing pathological synergies. A hallmark feature across all four of these conditions is the reduced flexibility in adapting available muscle synergies to task requirements. In stroke and spinal cord injury, this is readily seen by the casual observer. In focal hand dystonia, the reduction in flexibility is restricted to one or two tasks, at least early in the course of the disorder. In CTS, the reduced flexibility is far less obvious and has only become apparent with highly quantitative measures of movement. A weakness of our theoretical framework for dystonia and CTS is that the conditions are multifactorial and pathological synergies are inconsistent across subjects.

A difference that emerges across the four conditions is regarding how reflexive activity contributes to the pathological synergies. In stroke and spinal cord injury, hyperactive reflexes are present and may play a role in the observed stereotypic synergies. In contrast, focal hand dystonia and CTS tend to be associated with decreased, or hypoactive reflexes. Thus, the reflexive component is a piece of pathological synergies in some conditions but not others.

Across all four conditions, pathological synergies and the observed movement behaviors are affected by other factors and, in turn, affect other parts of the movement system. Two examples are described here and are not intended to be an exhaustive list. For stroke, pre-existing comorbidities, such as diabetes and hypertension, influence recovery (Go et al., 2013). After the initial lesion, the pathological synergy has downstream effects on muscle tissue and joint range of motion (Jakobsson et al., 1991, 1992; Gemperline et al., 1995; Given et al., 1995; Frontera et al., 1997). For focal hand dystonia, genetic predisposition or previous trauma can influence the severity and course of the condition (Altenmuller and Jabusch, 2010; Jinnah et al., 2013). On the downstream side, sustained postures often result in shortened muscles and excessive activation.

The neurobiological underpinings of each condition also provide insight into the opportunity to “recover” normal synergies. For pathological synergies in stroke and spinal cord injury, where there is a substantial loss of neurons, it may be exceedingly difficult to move in any way except for the abnormal synergistic pattern. In contrast, education and specifically tailored exercises for people with CTS can alter the gripping pattern. Going further, decompression and return of sensory and motor nerve conduction may eliminate the pathological synergy altogether.

## Directions for Future Research

While there has been tremendous growth in our understanding of physiological and pathological movement synergies, there is still a great deal left unknown. A major need for future research is large, longitudinal studies of synergies in various patient populations. As discussed in the opening sections of this review, physiological synergies are complex because they blend a “hard-wired” component (i.e., biomechanical constraints such as tendons spanning more than one joint) and a “soft-wired” component (flexible interaction of sensory information and activation of specific combinations of muscles). The presence of intact, flexible, and modifiable physiological synergies are what allows human beings to function so successfully in our changing environments. In order to re-establish a flexible physiological synergy versus an abnormal fixed synergy after nervous system injury, one needs to track if and how this can occur over long periods of time, including how synergistic control might or might not respond to various interventions.

One possibility that might yield a better understanding of pathologic synergies would be to develop a classification system based on synergy severity. An example of such an approach is provided by Cheung et al. (2012) who associated specific patterns of muscle coordination with the severity of functional impairment and the time from stroke onset. A classification system of synergy severity might be most logically developed within a given condition and not across conditions. Such a system that might be very useful for categorizing people into various subpopulations could be tested and eventually may respond differently to specific interventions. An argument against the utility of a severity classification scheme is that pathological synergies may not be the most relevant aspect of the condition for recovery. For example, work from Dr. Lang and others would argue that it is paresis and not abnormal synergies that are the greatest contributor to functional loss after stroke (Bohannon and Smith, 1987; Bourbonnais and Vanden Noven, 1989; Mercier and Bourbonnais, 2004; Lang and Beebe, 2007; Beebe and Lang, 2008, 2009). Likewise, it would be reasonable to argue that pain and loss of somatosensation could be the main drivers of functional deficits in CTS (Zhang and Santello, 2014). Thus, the effort of grading and categorizing could be an interesting scientific exercise, but in the long run could turn out to be wasted effort in the quest for better outcomes for people affected by these conditions. It is too early to tell, at the present time, which side of this argument will be the eventual winner.

In order to improve our understanding regarding how to rehabilitate patients with abnormal, fixed synergies, we must be able to quantify pathological synergies in a more standardized fashion within and across patient populations. An ideal standard methodology would be flexible so that it was sensitive to the idiosyncratic features of a range of conditions (e.g., from focal hand dystonia to CTS) and a range of severities (e.g., from mild to severe). Furthermore, the methodology should be simple, so that it could be incorporated into clinical care (e.g., grip devices that measure coordination of finger forces and not just maximum force across all fingers). Incorporation into clinical care would permit efficient data collection on larger, more heterogeneous patient samples that can be followed over long periods of time. Mechanical, electronic, and computational technologies are advancing so rapidly that one can easily envision clever, non-obtrusive devices that can quantify synergistic control in the not-so-distant future.

Ongoing research to cure stroke, focal hand dystonia, spinal cord injury, and CTS is progressing. These cures can take many different forms, such as novel pharmaceutical agents, stem cell implantations, and implanted or external biomedical devices. The eventual success of these potential future cures will depend on their ability to restore flexible, adaptable movement synergies. Some important questions that need to be addressed include “How many synergies are needed for function?” “Which synergies are most important to restore?” “How flexible does a synergy need to be for effective use in one’s daily environment?” and “How does one most efficiently retrain synergies?” A logical approach would be to start perusing these questions now, so that as potential cures emerge from basic research labs, the appropriate motor behavioral training methods are in place to facilitate success.

## Conclusion

Physiological synergies allow for repeatable, yet flexible coordination of multi-joint movements that can adapt to changing environments. Normal physiological synergies in persons with an intact nervous system can become pathological synergies when damage occurs at various levels of that system. Understanding the pathological synergies, whether or not they are amenable to change, or the extent to which they can be modified, and how they affect a person’s ability to move flexibly in daily life is important for healthcare providers, such as neurologists, physiatrists, physical therapists, occupational therapists, and nurses. As reviewed, various neurological conditions disrupt synergistic control at different points within the nervous system (Figure 2) with different effects on movement control. To better understand pathological synergies and improve the chances of restoring normal physiological synergies, longitudinal studies with large sample sizes and standard quantitative methodology are needed, together with efforts aimed at elucidating the mechanisms through which the CNS exploits neural and biomechanical constraints for controlling multi-degrees of freedom movements.

## Conflict of Interest Statement

The authors declare that the research was conducted in the absence of any commercial or financial relationships that could be construed as a potential conflict of interest.
